# Functional diversity of plant communities and species diversity in response to soil factors at different successional stages in karst landscapes

**DOI:** 10.3389/fpls.2025.1688827

**Published:** 2025-11-12

**Authors:** Yang Wang, Hong Huang, Yangyang Ji, Ruiyu Zhou, Yi Liang, Zhifeng Chen, Yao Lv, Juan Tao, Li Li

**Affiliations:** 1 School of Ecological Engineering, Guizhou University of Engineering Science, Bijie, China; 2 Guizhou Key Laboratory of Plateau Wetland Conservation and Restoration, Bijie, China

**Keywords:** succession, species diversity, functional diversity, soil factors, karst

## Abstract

Karst plant communities are significantly influenced by habitat heterogeneity. Investigating the effects of species diversity and functional diversity on soil properties is essential for the restoration and conservation of forest ecosystems. Using plant communities at various successional stages in the Doupeng Mountain area of Guizhou Karst, we applied one-way ANOVA, network correlation analysis, redundancy analysis, and structural equation modeling to assess the impact of soil factors on species and functional diversity, as well as the relationships between these diversity metrics, based on data from community surveys. The results showed that (1) The Simpson, Shannon, Pielou, and Margalef species diversity indices were significantly higher in the tree stage than in the grass and shrub stages. (2) Functional richness and the Rao coefficient differed significantly across successional stages of plant communities and were highest in the tree stage, whereas functional divergence varied significantly among stages and was highest in the grass stage. (3) As succession progressed, the correlations between species diversity, functional diversity, and soil factors gradually strengthened. Five soil factors—soil nitrogen-to-phosphorus ratio, soil carbon-to-nitrogen ratio, soil bulk density, soil phosphorus content, and soil organic matter—had significant effects on the species diversity index (*P* < 0.05). Similarly, these five soil factors significantly influenced the functional diversity index (*P* < 0.05). Additionally, soil phosphorus content, soil carbon-to-nitrogen ratio, soil nitrogen-to-phosphorus ratio, and soil carbon-to-phosphorus ratio were significant factors affecting both community species diversity and functional diversity. This study demonstrated that species diversity and functional diversity of communities at different successional stages in karst landscapes differed significantly and were influenced by soil nutrient content and nutrient allocation.

## 1 Introduction

Species diversity and functional diversity are crucial components in studying ecosystem functioning (Wang K. et al., 2022). Species diversity reflects the richness of species composition within plant communities, whereas functional diversity illustrates the response mechanisms and distribution of plant functional traits in relation to the environment (Petchey and Gaston, 2002; Han et al., 2021). Investigations of species and functional diversity can reveal ecosystem responses to environmental changes at the community level, serving as key factors in understanding plant fitness strategies and predicting ecosystem functions (Pan et al., 2021). Along different environmental gradients, species diversity and functional diversity exhibit varying patterns influenced by environmental factors such as climate, topography, and soil. A consensus on the universal relationship between species diversity and functional diversity has not yet been established (Xiang et al., 2019; Liu et al., 2021). Determining the distribution ranges of species traits closely linked to ecosystem function within a community could provide a significant breakthrough in understanding the relationship between biodiversity and ecosystem function (Ali et al., 2017). Environmental factors can act as a “filter” that determines which species or traits can survive and persist within a community (Bruno et al., 2016). The combination of multiple ecological factors creates different habitat types, and habitat heterogeneity is a crucial factor in maintaining species diversity (Zhao et al., 2007).

Community succession is not a process of single-species replacement but rather the result of numerous functional traits interacting to adapt to the environment (Chai et al., 2016). Using functional traits instead of species identity is more effective in revealing the drivers of succession and clarifying the ecological strategies of plants at different successional stages (Kahmen and Poschlod, 2010). The relationship between species diversity and functional diversity is complex and variable, influenced by environmental resources and external disturbances. Liu et al. (2013) found a positive correlation between species diversity and functional diversity, with the strength of this correlation increasing as precipitation decreased. They also observed positive correlations among meadow grassland, typical grassland, and meadowland. During karst vegetation restoration, significant differences exist in the composition and structure of plant communities across various restoration stages. It is generally accepted that increased species diversity is accompanied by enhanced functional diversity, reflecting greater community stability and resilience to disturbance as succession progresses (Dietrich et al., 2024; Yu X. et al., 2021). However, other studies have found that competition among species can lead to the loss of species possessing certain functional traits within the community, resulting in a negative correlation between species diversity and functional diversity (Lambers et al., 2011). Bu et al. (2014) reported that the relationship between species diversity and functional diversity across the entire successional sequence follows an S-shaped curve, indicating some functional redundancy during the early and late stages of succession. Species diversity is central to biodiversity, providing an intuitive measure of the richness of regional biological resources (Wei et al., 2014). It has been demonstrated that community functional diversity plays a crucial role in the complementary utilization of forest ecosystem resources, resistance to biological invasions, enhancement of forest productivity, and maintenance of biodiversity (de Bello et al., 2007). Recently, numerous scholars have conducted extensive research on the coupling relationship between species diversity and functional diversity. Exploring this coupling can help elucidate the mechanisms underlying community species coexistence and the stability of ecosystem services. The study of the “species diversity–functional diversity” coupling relationship further aids in understanding the processes that sustain community species coexistence and ecosystem service stability (Xu et al., 2019). Therefore, the core scientific questions of this study are: (1) How do species diversity and functional diversity vary along a karst ecosystem succession gradient? (2) Which soil factors play a dominant role in regulating these relationships? Investigating the characteristics of species and functional diversity under varying environmental conditions, as well as their interrelationships, can deepen and broaden our understanding of community diversity and reveal the adaptive mechanisms of plant communities in response to environmental changes.

## 2 Materials and methods

### 2.1 Study area description

Duyun City is located in the southeastern part of Guizhou Province, China, between longitudes 107°07′19″E–107°46′26″E and latitudes 25°51′26″N–26°25′39″N. The sample area is located within the eastern subtropical evergreen broad-leaved forest zone of China, characterized by uniformly distributed soil thickness. Detailed information about the sample site is provided in **Table 1**. The Duyun Doupeng Mountain Reserve in Guizhou lies within the broad-leaved evergreen forest region of eastern subtropical China. The reserve exhibits significant altitudinal variation, predominantly limestone geology, uneven soil thickness distribution, and moist soils, creating favorable conditions for plant growth across different elevations. Consequently, the forests in this area consist primarily of natural vegetation with high originality and diverse forest types, including evergreen broad-leaved forests, evergreen-deciduous mixed forests, deciduous broad-leaved mixed forests, deciduous broad-leaved forests, and broad-leaved mixed forests. Additionally, the region contains coniferous forests, coniferous-broad-leaved mixed forests, and bamboo forests. The average annual rainfall is 1,431.1 mm, and the average annual temperature is 16.0°C.

**Table 1:** Basic information on the study area.

Stages	X(°)	Y(°)	Elevation (m)	Dominant species
CD1	107.379101	26.375435	1177.6	*Miscanthus sinensis Anderss*
CD2	107.377389	26.37764	1159.4	*Miscanthus sinensis Anderss, Saxifraga stolonifera Curt*
CD3	107.377294	26.37727	1159.2	*Nephrolepis auriculata (L.) Trimen, Miscanthus sinensis Anderss*
GM1	107.475132	26.383977	929.33	*Lithocarpus glaber (Thunb.) Nakai, Rhus chinensis Mill., Ligustrum lucidum Ait.*
GM2	107.449988	26.372676	872.57	*Viburnum fordiae Hance, Lithocarpus glaber (Thunb.) Nakai, Pittosporum brevicalyx (Oliv.) Gagnep.*
GM3	107.506276	26.351394	831.46	*Symplocos congesta Benth., Pittosporum brevicalyx (Oliv.) Gagnep.*
QM1	107.544969	26.355319	863.3	*Pittosporum brevicalyx (Oliv.) Gagnep., Cunninghamia lanceolata (Lamb.) Hook., Machilus pingii Cheng ex Yang*
QM2	107.5069	26.35304	834.78	*Cunninghamia lanceolata (Lamb.) Hook., Liquidambar formosana Hance*
QM3	107.50147	26.351435	831.11	*Lindera communis Hemsl., Machilus pingii Cheng ex Yang, Cunninghamia lanceolata (Lamb.) Hook.*

CD, herbaceous stage plant community; GM, shrub stage plant community; QM, tree stage plant community.

### 2.2 Sample plot setting and survey

In September and October 2024, we conducted a community survey in the Doupeng Mountain Reserve, Duyun City, Guizhou Province, China, using a sampling method based on the principles of homogeneity, accessibility, and reasonableness (**Table 1**). To prevent any intentional bias in the samples and research findings, we employed random sampling methods to establish the study plots. We established three 30 m × 30 m tree plots, three 20 m × 20 m shrub plots, and three 20 m × 20 m herbaceous plots. Each tree plot was subdivided into nine 10 m × 10 m subplots. For the shrub plots, four 5 m × 5 m subplots were established at the four corners. In the herbaceous plots, five 1 m × 1 m subplots were set up at the four corners and the center of each plot. Quantitative data were recorded for each plant in the tree, shrub, and herb layers, including species name, diameter at breast height (DBH), plant height, and crown width (Wang Y. et al., 2022). Quantification of tree layer vegetation data included the following: (a) measure breast height diameter using a tape measure (brand: NEWBIES, accuracy: ± 1mm), taking readings approximately 1.3 m above ground level; (b) determine maximum horizontal crown projection widths in east–west and north–south directions using a laser rangefinder. Measurements were taken using a laser rangefinder. The procedure involved ensuring clear visibility of both the tree’s crown and base, aligning the instrument with the base of the trunk, then moving it upward to the apex of the crown before reading the displayed measurement (laser rangefinder brand: Shendawei, model: SW-600A, accuracy: ± 0.5 m, origin: Guangdong, China). Quantification of shrub layer vegetation included the following: measure the basal diameter of shrub species using a tape measure at approximately 0.1 m above ground level, employing the same tape measure parameters as for tree measurements. Canopy cover and shrub height measurements follow the same methodology as for the tree layer. Quantification of herbaceous layer vegetation is as follows: Herbaceous plant height is measured using a tape measure. Cover estimation employs visual assessment within 1 m × 1 m sample plots (as counting individual plants would entail excessive, meaningless labor).

### 2.3 Measurement of soil factors

Eight indicators were selected as soil factors: soil bulk density (BD)(BD = dried soil mass (g)/soil volume per unit area (cm³)), soil water content (SWC) (SWC = (weight of soil in natural state - weight of dried soil) (g)/weight of dried soil (g) × 100%), soil organic matter (SOC), soil total nitrogen (SNC), soil total phosphorus (SPC), soil carbon-to-nitrogen ratio (SCN), soil carbon-to-phosphorus ratio (SCP), and soil nitrogen-to-phosphorus ratio (SNP). The five-point sampling method was employed, collecting samples from the corners and center of each plot. Soil samples were excavated to a depth of 15–20 cm; part of the sample was placed in aluminum boxes, whereas the remainder was stored in self-sealing bags, labeled, and transported to the laboratory for natural drying. The original weight of the soil in the aluminum boxes and the weight after drying were measured to calculate soil bulk density and soil moisture content. The bagged soil samples were ground and sieved through 60-mesh and 100-mesh sieves, and 100 g was weighed for chemical analysis. Soil organic matter was determined using the potassium dichromate–sulfuric acid oxidation method; soil total nitrogen was measured by the Kjeldahl method; and soil total phosphorus was analyzed using the sodium hydroxide alkali fusion-molybdenum antimony colorimetric method (Bao, 2005).

### 2.4 Functional trait selection and measurement

Seven indicators—leaf thickness, leaf area, leaf dry matter mass, specific leaf area, chlorophyll content, leaf aspect ratio, and leaf tissue density—were selected as functional traits and used as the basis for calculating functional diversity. For the determination of leaf traits, fresh mass was first measured using an electronic balance (BSM-220.4, Shanghai Joujing Electronic Technology Co., Ltd., Shanghai, China) with an accuracy of 0.0001 g. Subsequently, the leaf area (LA, cm²) of each leaf was measured using a leaf area meter (Yaxin-1241, Beijing Yaxin Instrument Technology Co., Ltd., Beijing, China). The leaf area was then divided into two fractions using a portable chlorophyll content meter (SPAD-502 Plus, Konica Minolta, Tokyo, Japan). Leaf thickness (LT, mm) and chlorophyll content (CHL, SPAD) were also measured with the same portable chlorophyll content meter. After these measurements, the leaves were placed in numbered envelopes and dried in an oven at 80°C for 72 h until a constant mass was achieved. The dry mass of the leaves (LDM, g) was then weighed and recorded. Specific leaf area (SLA, cm²/g), leaf dry matter content (LDMC, mg/g), and leaf tissue density (LTD, g/cm³) were calculated based on the ratios of leaf area to leaf dry mass, leaf dry mass to fresh leaf mass, and leaf dry mass to leaf volume (calculated as leaf area multiplied by leaf thickness).

### 2.5 Species diversity and functional diversity calculations

The Shannon–Wiener diversity index (H), Pielou evenness index (J), Simpson diversity index (D), and Patrick richness index (R) were used as measures of community species diversity. The formulas for these indices are presented in **Table 2**.

**Table 2:** Species diversity and functional diversity formula.

Diversity type	Diversity index	Formula	Property specification
Species diversity	Shannon-Winener	$H=-\sum_{i=1}^{S}P_{i}\ln P_{i}$	S is the number of species; P*_i_* is the relative abundance of the ith species, P*_i_*= N*_i_*/N; N is the sum of the individual numbers of all species; N*_i_* is the total number of individuals of the ith species
	Pielou	J=H/lnS	
	Simpson	$D=1-\sum_{i=1}^{S}P_{i}^{2}$	
	Patrick	R=S	
Functional diversity	Functional richness	$FRic=\frac{SFci}{Rc}$	SFci is the niche occupied by species in community *i*, and Rc is the niche occupied by character c in community.
	Functional uniformity	$FEve=\frac{\sum_{i=1}^{S-1}min=\left(PEW_{i},\frac{1}{S-1}\right)-\frac{1}{S-1}}{1-\frac{1}{s-1}}$ $PEW_{i}=\frac{EW_{i}}{\sum_{i=1}^{S-1}EW_{i}}$ $EW_{i}=\frac{dist\left(i,j\right)}{n_{i}+n_{j}}$	*i* is the branch length, PEWi is the weight of the branch length, EW*_i_* is the uniformity weight, dist (i, j) is the Euclidean distance between species *i* and *j*, and n*_i_* represents the number of species *i*
	Functional divergence	$FDiv=\frac{\delta d+\bar{dG}}{\delta \left|d\right|+\bar{dG}}$ $\delta d=\sum_{i=1}^{S}P_{i}\times \left(dG_{i}-\bar{dG}\right)$	dG*_i_* is the Euclidean distance, dG is the mean of the Euclidean distance of each species character, δ| d |It is obtained by taking the absolute value of the distance difference when calculating δd, and P*_i_* is the relative abundance of the ith species.
	Rao’s quadratic entropy index	$RaoQ=\sum_{i=1}^{S-1}\sum_{i=i+1}^{S}d_{ij}p_{i}p_{j}$	p*_i_* is the proportion of species *i* to the total number of individuals in the community

### 2.6 Data processing

Data were initially organized using Microsoft Excel 2022. Prior to analysis, the data were tested for normality and subjected to ANOVA. Statistical analyses were conducted using SPSS version 31.0, including one-way ANOVA and Tukey’s HSD test for multiple comparisons of species diversity and functional diversity across different successional stages. Visualization and further analyses were performed in R (version 4.3.2) using various packages, including “patchwork”, “ggplot2”, “ggpubr”, “ggsci”, “grDevices”, “ggsignif”, and “reshape2.” Redundancy analysis of species diversity and functional diversity in relation to soil factors was conducted using the “vegan”, “ggplot2”, “ggrepel”, “ggsci”, and “rdacca.hp” packages. Additionally, redundancy analysis of soil factor evolution was performed using the “WGCNA” and “igraph” packages. Network analyses of species diversity, functional diversity, and soil factors at different successional stages were also carried out with “WGCNA” and “igraph”. Bubble diagrams were generated using “reshape2” in combination with “ggplot2”. Structural equation modeling of species diversity, functional diversity, and soil factors was performed using Amos 26.0. Graphs were finalized using Adobe Illustrator 2022.

## 3 Results

### 3.1 Species diversity and its variation across different successional stages

**Figure 1:**
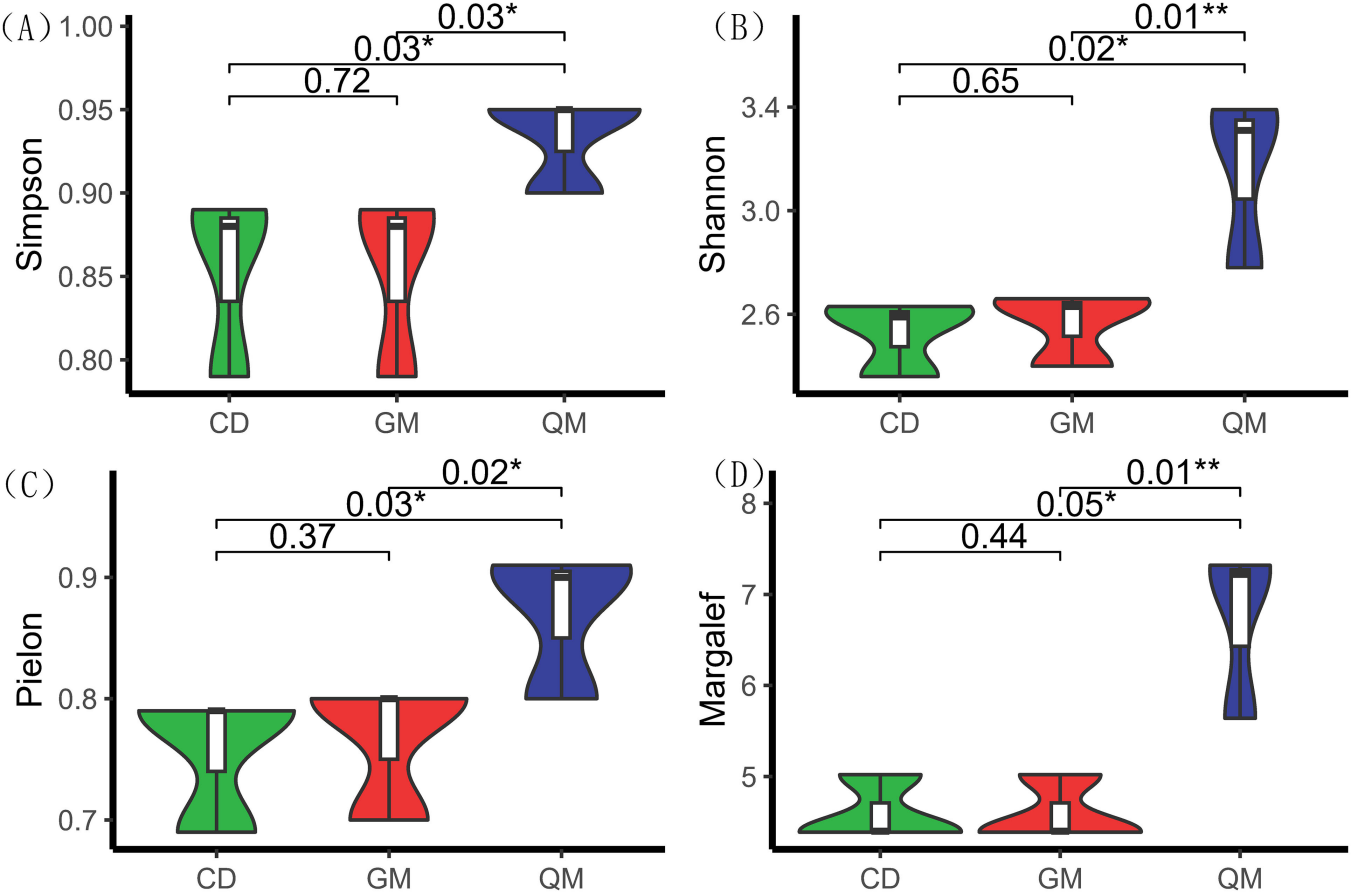
Species diversity of plant communities at different successional stages. One-way ANOVA was used for comparative analyses. * indicates significant differences (*P* < 0.05), and ** indicates highly significant differences (*P* < 0.01). The horizontal axis represents different stages of plant community succession, with abbreviations as per **Table 1**. The vertical axis represents species diversity indices, with index definitions as per **Table 2**. **(A)** denotes Simpson's diversity index, **(B)** denotes Shannon's diversity index, **(C)** denotes species evenness index, and **(D)** denotes species richness.

According to **Figure 1**, the diversity indices—Simpson, Shannon, Pielou, and Margalef—were significantly higher in the tree stage than in the grass and shrub stages. Among these indices, no significant differences were observed between the grass and shrub stages, whereas significant differences were found between the shrub and tree stages. This indicates that the transition from the shrub to the tree stage is more pronounced in plant community succession.

### 3.2 Functional diversity and its differences at different successional stages

**Figure 2:**
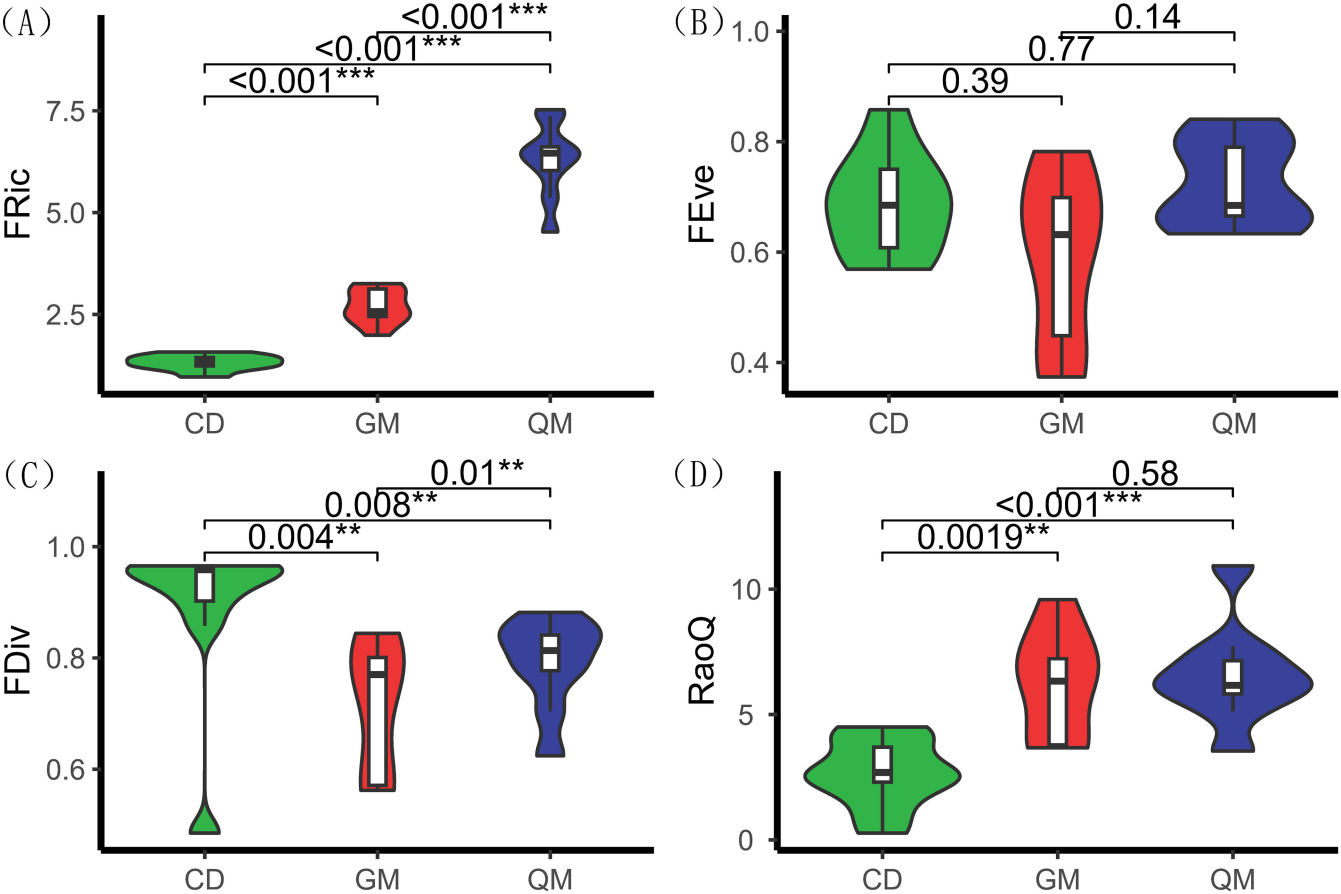
Functional diversity of plant communities at different successional stages. One-way ANOVA was used for comparative analyses. ** denotes highly significant differences (*P<* 0.01), and *** denotes extremely significant differences (*P* < 0.001). The horizontal axis represents different stages of plant community succession, with abbreviations referenced in **Table 1**. The vertical axis represents functional diversity indices, with index definitions referenced in **Table 2**. **(A)** denotes functional richness, **(B)** denotes functional evenness, **(C)** denotes functional dispersion, and **(D)** denotes Rao's coefficient.

According to **Figure 2**, functional richness differed significantly across various successional stages of plant communities, particularly during the tree stage. The Rao coefficient and functional richness index followed the same trend, showing significant differences across successional stages: tree stage (7.17) > shrub stage (6.05) > grass stage (3.38). Functional divergence also exhibited significant differences among stages, with values of grass stage (0.92) > tree stage (0.81) > shrub stage (0.72). However, functional homogeneity did not show significant differences across the different successional stages of the plant community.

### 3.3 Association of diversity with soil factors at different successional stages

**Figure 3:**
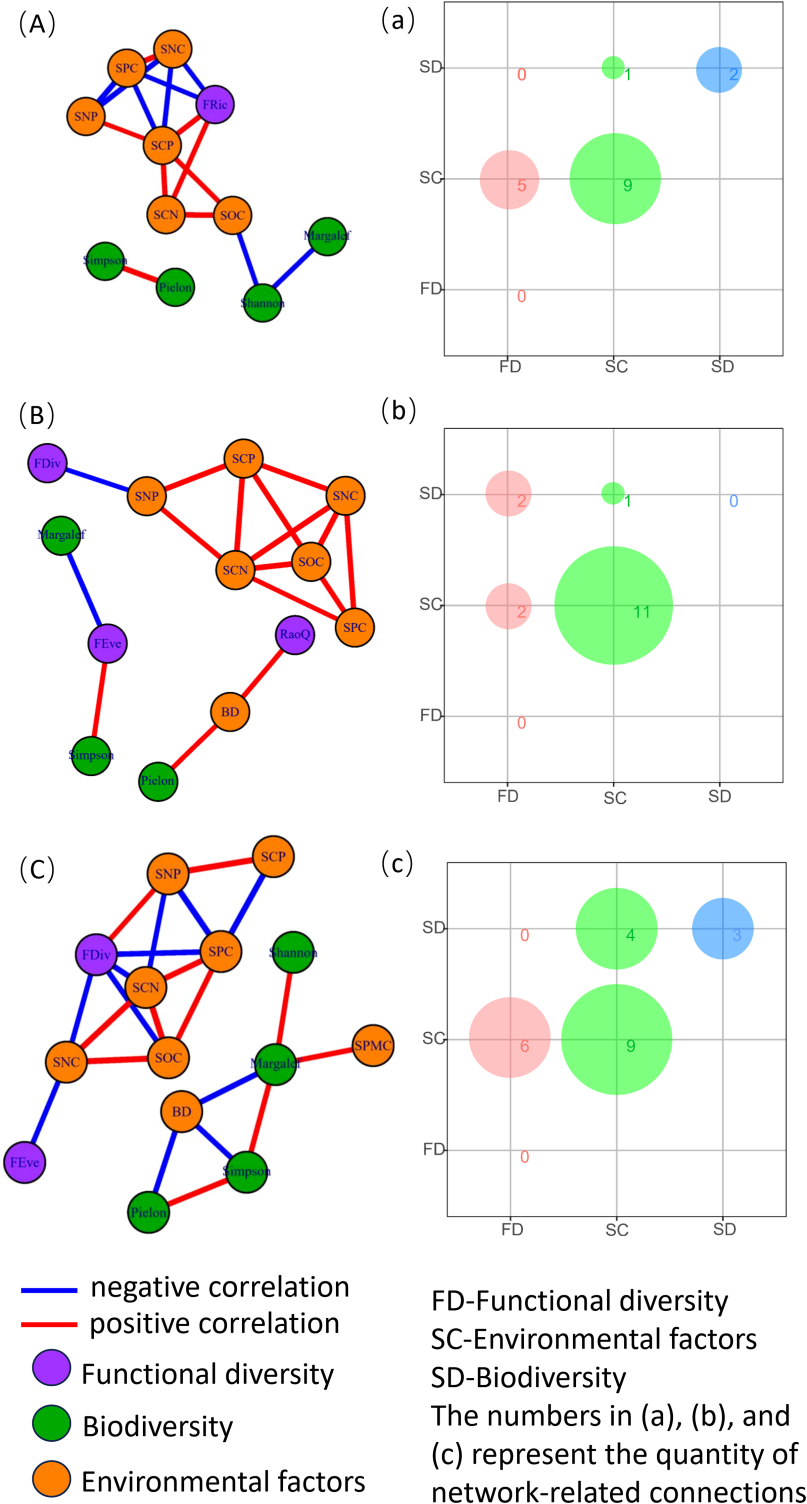
Species diversity, functional diversity, and soil factor network analysis across different successional stages of plant communities. Panels **(A, a)** represent the grass stage, **(B, b)** represent the shrub stage, and **(C, c)** represent the tree stage of the plant community. BD, soil bulk density; SWC, soil water content; SOC, soil organic carbon content; SNC, soil total nitrogen content; SPC, soil total phosphorus content; SCN, soil carbon-to-nitrogen ratio; SCP, soil carbon-to-phosphorus ratio; and SNP, soil nitrogen-to-phosphorus ratio.

According to **Figure 3**, during the grass phase, there were nine association line segments between soil factors, two between species diversity, five between functional diversity and soil factors, and one between species diversity and soil factors. In the shrub stage, there were 11 association line segments between soil factors, 2 between functional diversity and soil factors, 1 between species diversity and soil factors, and 2 between species diversity and functional diversity. In the tree stage, there were nine association line segments between soil factors, three between species diversity, six between functional diversity and soil factors, and four between species diversity and soil factors.

### 3.4 Influence of soil factors on species diversity and functional diversity

**Figure 4:**
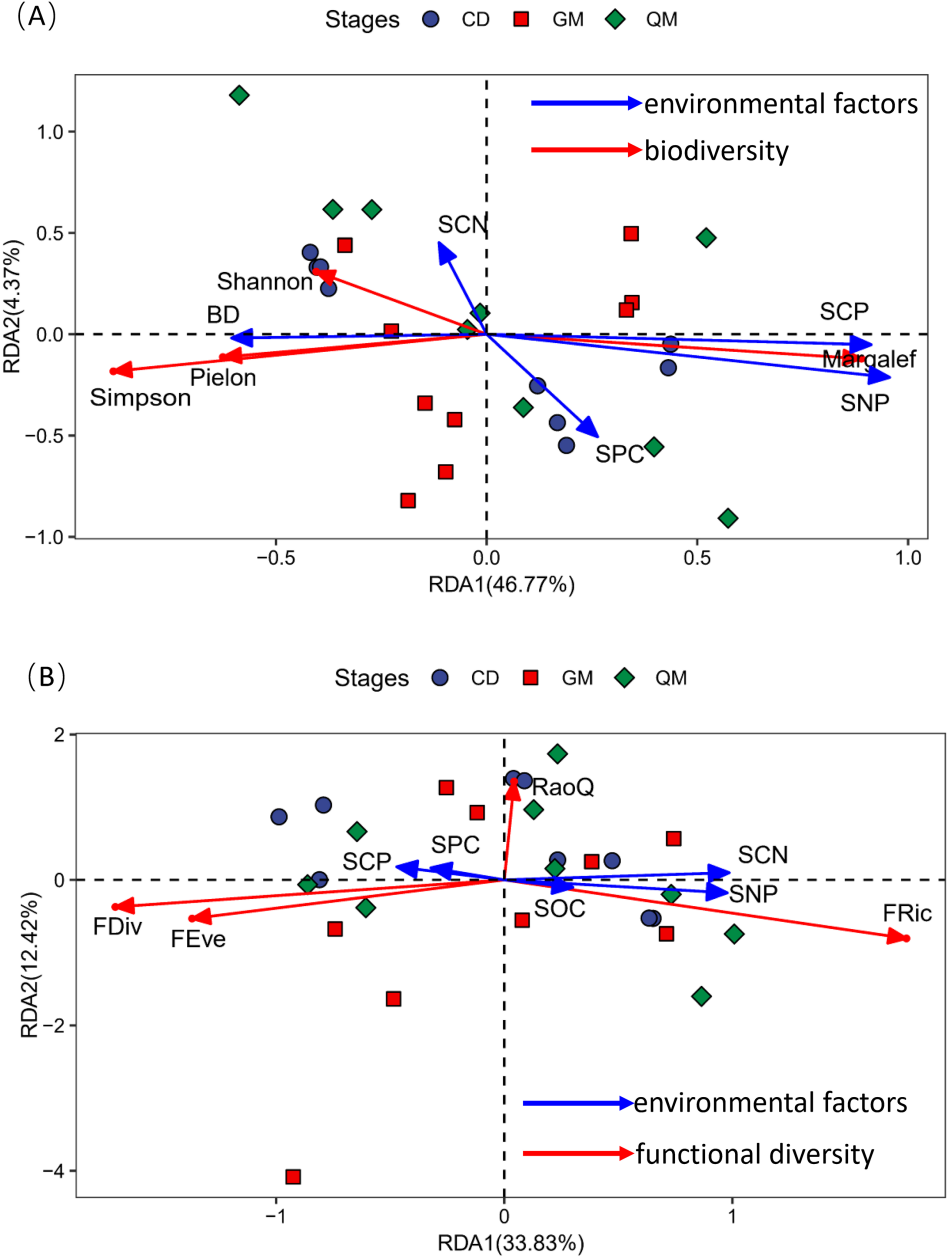
Redundancy analysis of community diversity in relation to soil factors. Panel **(A)** shows the redundancy analysis of species diversity with soil factors, whereas panel **(B)** illustrates the redundancy analysis of functional diversity with soil factors. The abbreviations for species diversity indices and functional diversity indices are referenced in **Table 2**; the abbreviations for environmental factors are referenced in **Figure 3**.

The DCA results showed that the maximum gradient lengths for community species diversity versus functional diversity and samples were 2.05 and 1.87, respectively. Therefore, the RDA linear model was selected for ordination to explore the relationship between plant community diversity and soil factors through redundancy analysis. In **Figure 4A**, the cumulative variance explained by the first two axes was 51.14%. The soil factors influencing plant community species diversity were ranked as follows: soil nitrogen-phosphorus ratio (SNP) > soil carbon-to-phosphorus ratio (SCP) > soil bulk density (BD) > soil phosphorus content (SPC) > soil carbon-to-nitrogen ratio (SCN). Additionally, species richness showed significant positive correlations with the soil nitrogen–phosphorus ratio and soil carbon-to-nitrogen ratio, whereas Simpson’s index, Shannon’s diversity, and species evenness showed significant positive correlations with soil water-holding capacity. In **Figure 4B**, the cumulative variance explained by the first two axes was 46.25%. The soil factors affecting the functional diversity of plant communities were ranked as soil nitrogen-phosphorus ratio (SNP)> soil carbon-to-nitrogen ratio (SCN)> soil carbon-to-phosphorus ratio (SCP)> soil phosphorus content (SPC)> soil organic matter (SOC). Furthermore, functional richness was significantly positively correlated with soil nitrogen-phosphorus ratio, soil carbon-to-nitrogen ratio, and soil organic matter. Functional evenness and functional divergence showed significant positive correlations with soil carbon-to-phosphorus ratio and soil phosphorus content.

**Figure 5:**
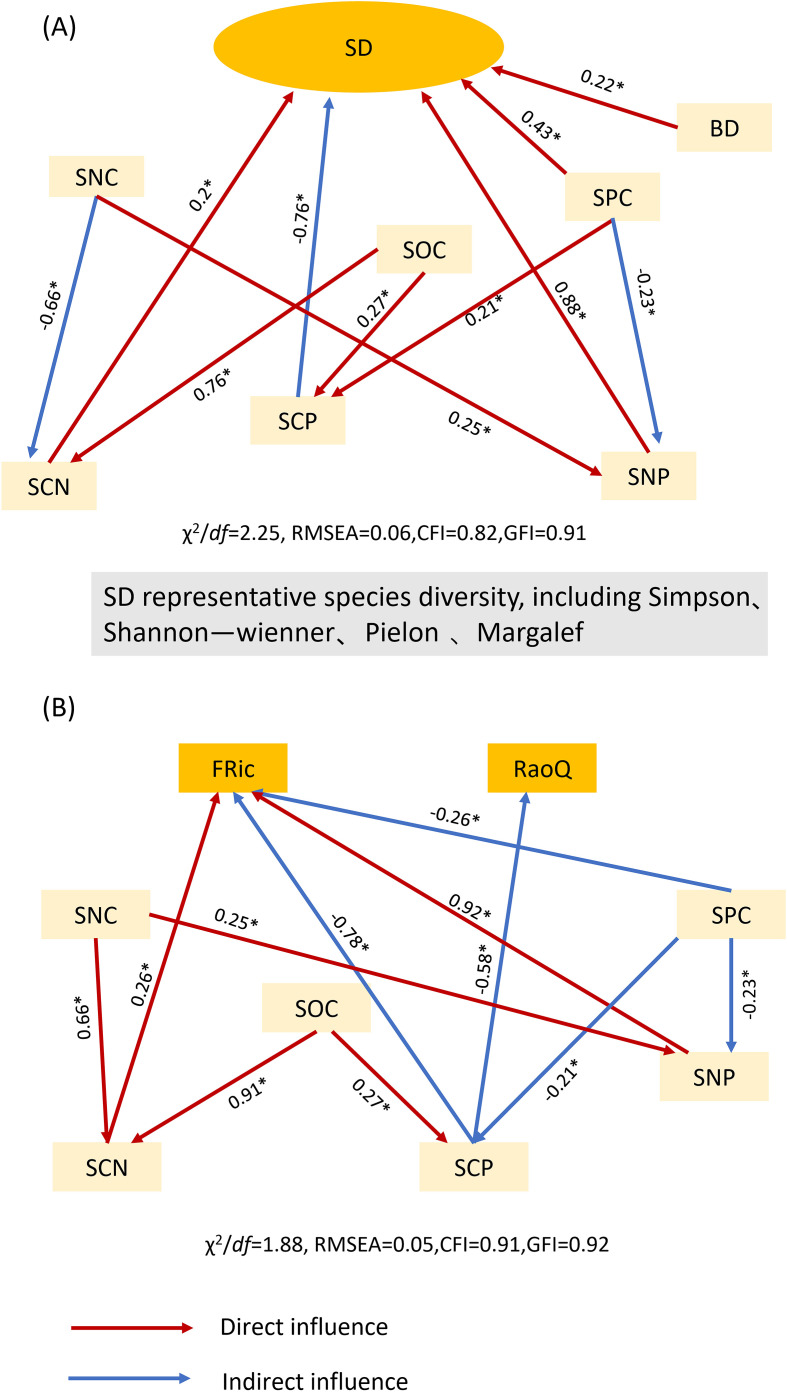
Structural equation modeling of soil factors and community diversity. **(A)** Structural equation modeling of soil factors and community species diversity; **(B)** structural equation modeling of soil factors and community functional diversity. The abbreviations for the functional diversity indices are listed in **Table 2**, whereas those for environmental factors are shown in **Figure 3**. χ² denotes the chi-squared test for goodness of fit; *df* denotes degrees of freedom; χ²/*df* denotes the ratio of chi-squared to degrees of freedom; GFI denotes the goodness-of-fit index; RMSEA denotes the root mean square error of approximation; and CFI denotes the comparative fit index. * denotes significant differences (P < 0.05).

To further elucidate the influence of soil factors on species diversity and functional diversity in plant communities, their correlation was analyzed using structural equation modeling (**Figure 5**). Functional evenness and functional divergence showed no significant effects in the model and were therefore excluded from the analysis. Instead, the functional richness index and Rao’s quadratic entropy were selected for evaluation. The results indicated that soil bulk density (BD), soil phosphorus content (SPC), soil carbon-to-nitrogen ratio (SCN), and soil nitrogen-to-phosphorus ratio (SNP) directly affected changes in species diversity. Additionally, the soil carbon-to-phosphorus ratio (SCP) indirectly influenced species diversity, with direct effects being stronger than indirect effects. Regarding functional diversity, the soil carbon-to-nitrogen ratio (SCN) and soil nitrogen-to-phosphorus ratio (SNP) had direct effects, whereas soil phosphorus content (SPC) and soil carbon-to-phosphorus ratio (SCP) exerted indirect effects, with indirect effects being stronger than direct effects.

## 4 Discussion

### 4.1 Characteristics of plant community diversity at different successional stages in karst landscapes

Species diversity is a crucial indicator of the complexity and stability of community ecosystems. Generally, higher species diversity corresponds to a more complex community structure (Hu et al., 2024). In this study, the number of species increased with succession, accompanied by a rise in the species diversity index, consistent with the findings of Yu et al (Yu et al., 2002). This suggests that in forest communities, species richness largely determines the species diversity index (Shang et al., 2023). Notably, the increase was more pronounced during the shrub stage compared with the tree stage, which may be related to nutrient differences and resource competition arising from habitat heterogeneity in the region. Furthermore, examining changes in plant community functional diversity essentially explores how plant communities occupy ecotone space and the distribution patterns of functional traits within that space (Mouchet et al., 2010; Fu et al., 2017). The functional richness index reflects the extent to which existing species occupy ecotone space; higher richness indicates more complete occupation, greater community productivity, and enhanced ecosystem stability (Tilman et al., 1997; Petchey and Gaston, 2002). The results of this study showed that functional richness (FRic) increased significantly with succession (**Figure 2**), suggesting that as succession progressed, the karst plant community’s resource occupation expanded.

Functional evenness measures the distribution of species traits within the occupied trait space and indicates the degree of resource utilization by the community (Barsoum et al., 2016). In this study, functional evenness (FEve) did not show significant differences between successional stages, suggesting that plant communities do not vary substantially in their efficiency of resource utilization, despite the heterogeneity of karst habitats. Functional divergence reflects the dispersion of community traits and is positively correlated with the level of ecological niche differentiation, while being inversely related to the intensity of interspecific resource competition (Mason et al., 2005). Here, the Rao coefficient was higher in the tree stage than in other stages, indicating that interspecific competition for resources within the plant community initially intensified and then weakened with succession. The degree of ecological niche differentiation increased from low to high, ecological niche overlap was enhanced, and functional redundancy increased after reaching the tree stage. The FRic index of the grassland community was significantly lower than that of shrub and tree communities, likely due to its lower species richness. This is attributed to the dominance of a few species in grassland communities, such as *Miscanthus sinensis Anderss, Saxifraga stolonifera Curt, and Nephrolepis auriculata (L.)*, which results in stronger intraspecific competition but weaker interspecific competition, leaving more available trait space within the community. Both the RaoQ functional diversity index and the species richness index (R) increased significantly with succession, suggesting ecological niche overlap in karst forest plant communities during succession. This indicates that interspecific competition shifted from intense to balanced and that the communities are undergoing positive succession (Hillebrand et al., 2008).

The results of this study (**Figure 3**) showed no significant relationship between functional diversity and species diversity at each successional stage of plant communities. This suggests that plant communities at different successional stages in the karst region exhibit relatively high functional diversity. A likely explanation for this finding is the increased habitat heterogeneity in the study area, which promotes variability in plant functional traits as species compete for ecological niches and resources. Furthermore, when considering the entire plant community (**Figure 4B**), functional evenness exhibited a strong positive correlation with functional divergence. This indicates that the spatial distribution of traits within the study area plays a crucial role in ecological niche differentiation and interspecific resource competition.

### 4.2 Species diversity and functional diversity in relation to soil factors

The diversity of plant communities is influenced by various environmental factors. Research has shown that topography and soil factors play a major role at small spatial scales, whereas elevation and climate factors are more influential at larger scales (Zu et al., 2019; Liang et al., 2023). This study focuses on plant communities at small scales, where soil factors predominantly affect ecosystems in karst regions characterized by high environmental heterogeneity. The results (**Figure 3**) indicate that, during the grassland stage, the correlation between species diversity and soil factors was 1/17, whereas the correlation between functional diversity and soil factors was 5/17. This may be because the structure and function of grassland ecosystems in the early successional stage are not severely impacted, and available soil nutrients remain relatively sufficient. Consequently, soil nutrient changes caused by degradation do not significantly affect species diversity (Peng et al., 2018). In the shrub stage, the correlation between species diversity and soil factors was 1/16, and the correlation between functional diversity and soil factors was 2/16. This suggests that the influence of soil factors on both species and functional diversity increases during this stage. This may be attributed to the deterioration of soil structure in the shrub stage, which leads to decreased porosity and reduced water permeability. Additionally, increased surface runoff results in nutrient loss, as reflected by changes in soil bulk density (Børgesen and Schaap, 2005). Furthermore, functional diversity shows a positive correlation with soil nitrogen-to-phosphorus (N:P) ratios. Plants require an appropriate nutrient balance for optimal uptake, and significant variations in soil nitrogen relative to other nutrients can impact plant growth and diversity (Güsewell, 2004). It has been suggested that imbalances in nitrogen–phosphorus ratios may alter plant community structure by promoting the growth of species tolerant to low nitrogen levels whereas suppressing those with higher nitrogen requirements, thereby affecting both community and functional diversity (Moore et al., 2019). During the tree stage, the correlation between species diversity and soil factors was 4/22, and the correlation between functional diversity and soil factors was 6/22. As succession progresses, plant community species diversity gradually increases, and stand structure becomes more complex. Concurrently, soil structure further deteriorates, aeration declines, and soil bulk density becomes more sensitive to soil water content (Hinsinger, 2001).

This study also demonstrated (**Figure 4A**) that, overall, Simpson’s index, Shannon’s index, and species evenness exhibited significant positive correlations with soil bulk density and the soil carbon-to-nitrogen ratio. Additionally, species richness showed significant positive correlations with the soil nitrogen-to-phosphorus ratio and the soil carbon-to-phosphorus ratio, consistent with findings from previous studies (Kuang et al., 2024). In ecosystems, soil and vegetation are interdependent and interact closely, with soil carbon, nitrogen, and phosphorus—key nutrient pools—playing critical roles in the system (Yu X. et al., 2021). Notably, this study found that the soil carbon-to-phosphorus ratio had a negative feedback effect on species diversity, whereas soil phosphorus content, the soil carbon-to-nitrogen ratio, and the soil nitrogen-to-phosphorus ratio positively influenced species diversity. Previous research has shown that nitrogen and phosphorus contents in karst areas are significantly lower than in other regions, and plant utilization rates of these nutrients are reduced (Liu et al., 2020). Furthermore, nitrogen intensifies competition for light resources, promoting the dominance of highly competitive species within plant communities and restricting the ecological niche space available to less competitive species through competitive exclusion. Such variations may result from differing types of nutrient limitation across ecosystems (Yahdjian et al., 2011). This study demonstrates a positive correlation between the C:N ratio and species diversity. Despite the shallow soil layers characteristic of karst regions, the topsoil exhibits high concentrations of elements. This phenomenon may result from the area’s high-temperature and high-humidity environmental conditions, which facilitate nutrient release from litter. The C:N ratio serves as an indicator for assessing the rate of soil organic matter decomposition. Within the study area, increased accumulation of organic matter enhances microhabitat heterogeneity, thereby providing greater resource availability for growth. Concurrently, diverse microhabitat types support soil microorganisms and small animals, exerting a strong effect on soil fertility (Zeng et al., 2016). Soil phosphorus is a key limiting nutrient for productivity in karst regions, directly contributing to biomass accumulation through its role in plant energy metabolism (Nasar et al., 2021; Yu Y. et al., 2021). This underscores the importance of soil nutrient partitioning in plant community succession within karst areas. Soil factors influence species diversity and productivity in karst plant communities through multiple pathways involving nutrient partitioning, nutrient balance, and biological processes (Nasar et al., 2021; Yu Y. et al., 2021; Tian et al., 2025). In this study, we demonstrated that soil nutrient partitioning is a central factor in maintaining species richness; however, the ecological effects of soil nutrients may be modified by changes in plant community succession.

In general, at small scales, elevation and relief are the two most important topographic factors influencing plant functional traits in South Asian tropical evergreen broadleaf forests, whereas soil water content and total nitrogen content are the most significant soil factors affecting these traits (Ding et al., 2011). However, this study demonstrated that functional uniformity and functional divergence exhibited significant positive correlations with soil phosphorus content and the soil carbon-to-phosphorus ratio. Additionally, functional richness showed significant positive correlations with soil organic carbon content, the soil nitrogen-to-phosphorus ratio, and the soil carbon-to-nitrogen ratio. Regarding soil factors, their relative importance was ranked as follows: soil nitrogen-to-phosphorus ratio > soil carbon-to-nitrogen ratio > soil phosphorus content > soil organic matter. Carbon, nitrogen, and phosphorus are essential nutrients for plant growth, and both their content and chemical forms in the soil significantly influence plant functional traits (Li et al., 2022). This study demonstrates that both soil phosphorus content and the C:P ratio exert negative feedback effects on functional diversity (**Figure 5B**). Phosphorus is a primary limiting nutrient in many forest regions worldwide, as severe soil phosphorus deficiency impairs certain photosynthetic processes (Wu et al., 2007). Research indicates that during succession, plant leaf thickness decreases whereas leaf area increases. These trait changes enhance photosynthesis, enabling plants to acquire resources more efficiently (Wang Y. et al., 2022). Although soil phosphorus content in this study did not show significant changes during succession, both C:P and N:P ratios exhibited significant variations. These differences lead to environmental selection that reshapes the functional traits and composition of plant communities at different succession stages due to assemblage effects driven by trait changes. These findings further confirm that karst plant communities are phosphorus-limited during succession. This study demonstrates some subjectivity in plot selection, and its methodology may have certain limitations. We aim to refine our research conclusions more comprehensively through future investigations that are more objective and scientifically rigorous.

## 5 Conclusion

The investigation of plant communities at three distinct successional stages—namely, grassland, shrubland, and forest—in the karst region revealed the following:

1. Species diversity and functional diversity of communities at different successional stages differed significantly, with the highest species richness observed in the tree stage and the lowest in the grassland stage. The relationship between community species diversity and functional diversity is complex, with a stronger correlation evident during the shrub stage.

2. Karst plant community diversity was jointly influenced by the soil nitrogen-phosphorus ratio, soil carbon-to-nitrogen ratio, and soil phosphorus content. This indicates that soil factors shape the species diversity and productivity patterns of karst plant communities through multiple pathways involving nutrient allocation, balance, and biological processes. Additionally, karst plant communities were limited by phosphorus availability during succession.

## Data Availability

The raw data supporting the conclusions of this article will be made available by the authors, without undue reservation.
